# Chronic Intake of Commercial Sweeteners Induces Changes in Feeding Behavior and Signaling Pathways Related to the Control of Appetite in BALB/c Mice

**DOI:** 10.1155/2018/3628121

**Published:** 2018-01-28

**Authors:** Alberto A. Barrios-Correa, José A. Estrada, Caroline Martel, Martin Olivier, Rubén López-Santiago, Irazú Contreras

**Affiliations:** ^1^Laboratorio de Neuroquímica, Facultad de Medicina, Universidad Autónoma del Estado de México, Paseo Tollocan s/n Esq. Jesús Carranza, Colonia Moderna de la Cruz, 50180 Toluca, MEX, Mexico; ^2^Laboratory of Host-Pathogen Interactions, Department of Microbiology and Immunology, McGill University Health Center, 1001 Boul. Décarie, Site Glen Pavilion E/Block E, Montréal, QC, Canada H4A 3J1; ^3^Laboratorio de Inmunología Celular I, Departamento de Inmunología, Escuela Nacional de Ciencias Biológicas, Instituto Politécnico Nacional, Prolongación de Carpio s/n, Santo Tomas, Miguel Hidalgo, 11340 Ciudad de México, Mexico

## Abstract

Nonnutritive sweetener use is a common practice worldwide. Although considered safe for human consumption, accumulating evidence suggests these compounds may affect metabolic homeostasis; however, there is no consensus on the role of frequent sweetener intake in appetite and weight loss. We sought to determine whether frequent intake of commercial sweeteners induces changes in the JAK2/STAT3 signaling pathway in the brain of mice, as it is involved in the regulation of appetite and body composition. We supplemented adult BALB/c mice with sucrose, steviol glycosides (SG), or sucralose, daily, for 6 weeks. After supplementation, we evaluated body composition and expression of total and phosphorylated JAK2, STAT3, and Akt, as well as SOCS3 and ObRb, in brain tissue. Our results show that frequent intake of commercial SG decreases energy intake, adiposity, and weight gain in male animals, while increasing the expression of pJAK2 and pSTAT3 in the brain, whereas sucralose increases weight gain and pJAK2 expression in females. Our results suggest that chronic intake of commercial sweeteners elicits changes in signaling pathways that have been related to the control of appetite and energy balance* in vivo*, which may have relevant consequences for the nutritional state and long term health of the organism.

## 1. Introduction

 Nonnutritive sweeteners are food additives that provide sweet taste without the energy content of sucrose. Use of these substances by the general population has increased greatly, as they are available in a variety of commercial products. The effect of sweeteners on appetite and energy balance in living organisms has been tested in both human and animal models; however, so far, there is no clear consensus on whether chronic intake of these additives has a beneficial or detrimental effect on these variables [1].

Evidence from animal models shows that supplementation with saccharin or aspartame, nonnutritive sweeteners that used to be common choices as food additives, increases appetite and adiposity compared to groups supplemented with glucose or sodium chloride, without altering food intake [2–4]. Similarly, results from human studies of aspartame supplementation have also shown an increase in daily energy intake, compared to subjects supplied with mineral water or sucrose [5]. In contrast, other studies have reported that supplementation with saccharin does not induce weight gain when compared to glucose [6], and it has also been reported that supplementation with a mixture of artificial sweeteners containing aspartame, cyclamate, acesulfame K, and saccharin, for 10 weeks in humans caused an average of 1.2 kg of weight loss, whereas those supplemented with 2 g/Kg/day of sucrose gained an average of 1.4 kg [7]. Finally, a report from human studies of supplementation with sucralose or steviol glycosides (SG), two of the most commonly used noncaloric sweeteners currently available, did not show differences in satiety or daily energy intake [8]. Therefore, there is no clear consensus on the effects of nonnutritive sweeteners on appetite and weight gain.

Appetite and energy balance are regulated by central and peripheral signals that interact to modulate the organism's nutrient intake. These mechanisms are mainly dependent on the functions of the hypothalamus, which obtains information about the nutritional state and energy availability in the organism through orexigenic and anorexigenic signals, including both central and peripheral hormones and neurotransmitters, among which leptin is considered one of the most relevant and is one of the most well-studied molecules involved in the control of appetite and energy balance [9]. Leptin acts through six membrane receptors (ObRa-f) with structural homology to type 1-cytokine receptors [10]. Similarly to cytokine receptors, the long isoform of the leptin receptor (ObRb) is able to activate the Janus kinase 2/signal transducer and activator of transcription 3 (JAK2/STAT3) signaling pathway [11]. The role of this signaling pathway in appetite, weight gain, and body composition has been well studied* in vivo*, as it is a key regulator of the anorexigenic effects of leptin by modulating the production of proopiomelanocortin (POMC), a molecule that has important implications in appetite and weight control, as the POMC-derived alpha-melanocyte-stimulating hormone (*α*-MSH) is a potent appetite suppressor and also increases energy expenditure [12, 13]. STAT3 also modulates the expression of thyrotropin releasing hormone (TRH), as well as locomotor activity and POMC cleavage, indirectly modulating appetite and energy expenditure [14–16]. In accordance with its role in the regulation of appetite and energy balance, murine models of impaired ObRb-STAT3 signaling within the CNS (*s/s *mice) exhibit similar characteristics to* ob/ob* and* db/db* mouse strains, which are obese, hyperphagic, and diabetic [17–20].

Although the signaling pathways involved in appetite control and energy balance are well known, there is no information regarding the effects of chronic, frequent sweetener intake on these processes, nor the molecular mechanisms involved. Since there is no consensus about the actual effect of sweetener consumption on appetite and energy balance, the present study aimed to assess the effects of supplementation with two of the most widely used noncaloric sweeteners currently available, sucralose and SG, on the expression of proteins involved in the JAK2/STAT3 signaling pathway in the brains of mice, following the hypothesis that chronic sweetener intake may alter the activity of this pathway, promoting changes in appetite and body weight.

## 2. Materials and Methods

Animal care and handling, dissections, and protein expression analysis were performed in the Faculty of Medicine, Universidad Autónoma del Estado de México, Mexico. Immunofluorescence analysis was performed at McGill University Health Center, Canada.

### 2.1. Animals and Experimental Groups

Male and female BALB/c mice were bred and raised under standard conditions (12 : 12 h light : dark cycle; 22°C constant temperature; access to food and water ad libitum). Mice were weaned at 3 weeks of age and segregated by sex. At 8 weeks of age, mice were randomly assigned to one of four study groups, comprised of 18 mice (9 male and 9 female) per group. Mice in all study groups were kept 3 per cage and supplied with specific quantities of rodent chow (5001, LabDiet Nutrition International) and purified water (100 mL), which were measured and replenished daily. Study groups were established as follows: (1) control (purified water); (2) sucrose (10% dilution of sucrose in 100 mL of purified water); (3) sucralose (one 1 g packet of commercial sucralose sweetener Splenda®, equivalent to 0.012 g of sucralose, in 100 mL purified water); and steviol glycosides (one 1 g packet of commercial SG sweetener Svetia®, equivalent to 0.025 g of SG in 100 mL purified water). All groups had free access to food and water. Dilutions used for commercial sweeteners were based on the equivalent dose used for human consumption and were previously determined not to cause aversion in our experimental animals. Sweetener supplementation was performed for 6 weeks. After supplementation, mice were sacrificed by intraperitoneal administration of sodium pentobarbital (50 *μ*l/25 g). Twelve brains from each experimental group (6 male, 6 female) were used for protein extraction and western blot analysis. The remaining 6 brains (3 male, 3 female) were used for immunofluorescence staining and confocal microscopy.

### 2.2. Food, Water, and Energy Intake and Determination of Weight Gain

Food and water intake were measured daily throughout the study. The mean quantity of food consumed by experimental animals was determined by measuring their daily food intake, based on administration of a known quantity of food for each cage (100 g) and the weight of food remaining 24 hr later. Results were divided by the amount of animals per cage (3 per cage) to obtain the mean value of daily food intake per animal. Water intake was determined by measuring the amount of liquid left 24 hr after supplying 100 mL of purified water or sweetener solution and dividing the results among the number of animals per cage. Energy intake was determined at the end of the study by adding the caloric content from food supplied, multiplying the amount of food consumed by the amount of calories per gram of chow stated by the manufacturer (4.09 Kcal/g) and calories consumed by drinking the sucrose solution (0.4 Kcal/g). The weight of each individual mouse was measured at the beginning of the experiment with a standard laboratory balance and then once every week until the end of the study.

### 2.3. Determination of Adiposity

Body composition was determined using a tetrapolar spectroscopy bioimpedance system (ImpediVet® Vet BIS1). Briefly, anesthetized rodents were placed facing down with the front paws out to the side and hind legs flat and spread backwards, allowing the placement of 4 needle electrodes to measure the impedance following the equipment's instructions.

### 2.4. Analysis of Protein Expression

After sacrifice, brains were dissected and the tissue disaggregated with lysis buffer (0.5 M Tris (pH 6.8), 0.2 M EDTA, 50 mM EGTA, 0.1% 2-mercaptoethanol, 1% IGEPAL, 20 *μ*g/mL aprotinin, 20 *μ*g/mL leupeptin, 1 mM PMSF, 2 mM Na_3_VO_4_, 50 mM NaF; Sigma Aldrich) at 4°C for 45 min, mixing by vortex every 15 min and centrifuged at 13,000 rpm, 25 min at 4°C. Supernatant was obtained and total proteins were quantified by the Bradford method (500-006, Bio-Rad), dosed and aliquoted with sample loading buffer (SLB; Tris-HCl, glycerol, 10% SDS, 2-mercaptoethanol, 1% bromophenol blue) and stored at –70°C for further use. 60 *μ*g of protein was separated by SDS-PAGE and transferred onto PVDF membranes (IPVH00010, Immobilon P) previously hydrated with 100% methanol. Membranes were blocked in 1% bovine serum albumin (BSA) in tris-buffered saline solution (TBS)-Tween 20, 1 h at room temperature, and incubated overnight at 4°C with primary antibodies against JAK2 (3230S), pJAK2 (3776S, detecting pY1007/1008), STAT3 (9132S), pSTAT3 (9131S, detecting pY705), Akt (9272S), and phosphorylated Protein Kinase B (pAkt) (9271S, detecting pS473). Antibodies were obtained from Cell Signaling Technology. Antibodies were titrated and used at 1 : 750 dilution in 5% BSA. Antibodies against SOCS3 (sc-7009, Santa Cruz Biotechnology) and ObRb (254537, Abbiotec) were used at 1 : 1000 dilution and incubated under identical conditions. After incubation with primary antibody, membranes were washed 3x 5 min with TBS-Tween 20 and incubated with a secondary goat anti-rabbit antibody (31460, Thermo Scientific) at 1 : 5000 dilution, except for membranes with SOCS3 antibody, which were incubated with secondary anti-goat IgG antibody (A5420, Sigma Aldrich) at 1 : 10000 dilution, for 1 hour at room temperature. After incubation, membranes were washed 3x 5 min with TBS-Tween 20 and developed with 150 *μ*L diaminobenzidine and 30 *μ*L hydrogen peroxide in 10 mL of phosphate-buffered saline solution (PBS). Loading control was performed using anti-actin mouse antibody at 1 : 10000 dilution (A4700, Sigma Aldrich). Densitometry analysis was performed using ImageJ software version 1.51f (NIH), with band density determined as arbitrary units (AU).

### 2.5. Analysis of ObRb Expression by Immunofluorescence

Whole brains were extracted and frozen in Optimal Cutting Temperature solution (OCT, Fisher) and stored at −70°C. 10 *μ*m thick coronal sections were cut and set onto previously cleaned glass slides. Sections were fixed 10 min at −20°C in acetone, rehydrated in PBS, and blocked with 1% BSA in PBS, 2 h at room temperature, in a wet chamber. Primary antibody against leptin receptor ObRb (1 : 1000) was incubated overnight at 4°C in 5% BSA with mild shaking. After incubation, slides were washed 3x 5 min with PBS and incubated with secondary goat anti-rabbit IgG Alexa Fluor 488-conjugated antibody (ab150077, Abcam) at 1 : 2000 dilution for 1 hour at room temperature in a dark chamber with mild shaking. Slides were washed 3x 5 min with PBS and mounted in Prolong Diamond Antifade Mountant with DAPI (36962, Thermo Fisher). Antibody specificity was determined by incubating samples with secondary antibody only. Slides were analyzed by confocal microscopy using a Zeiss LSM880 microscope. Optical fields containing a minimum of 25–40 cells were randomly obtained for each brain sample using sections cut at the brain's midline and analyzed for the presence of ObRb-positive cells. An approximate number of 150 cells were counted in each sample, obtaining total numbers between 260 and 470 total cells per group of male or female mice, and the frequency (%) of ObRb-positive cells was obtained and analyzed. Cell counting in each micrograph was performed with the Fiji image-processing package for ImageJ software version 1.51f (NIH).

### 2.6. Statistical Analysis

Data are expressed as mean ± SEM. Comparison of different groups was performed using differences between means, with a 95% confidence interval, and one-way analysis of variance for nonnormally distributed data, using SPSS ver.22 software (IBM). A value of *p* < 0.05 was considered statistically significant.

## 3. Results

### 3.1. Modifications of Feeding Behavior in Sweetener-Supplemented Mice

**Figure 1:**
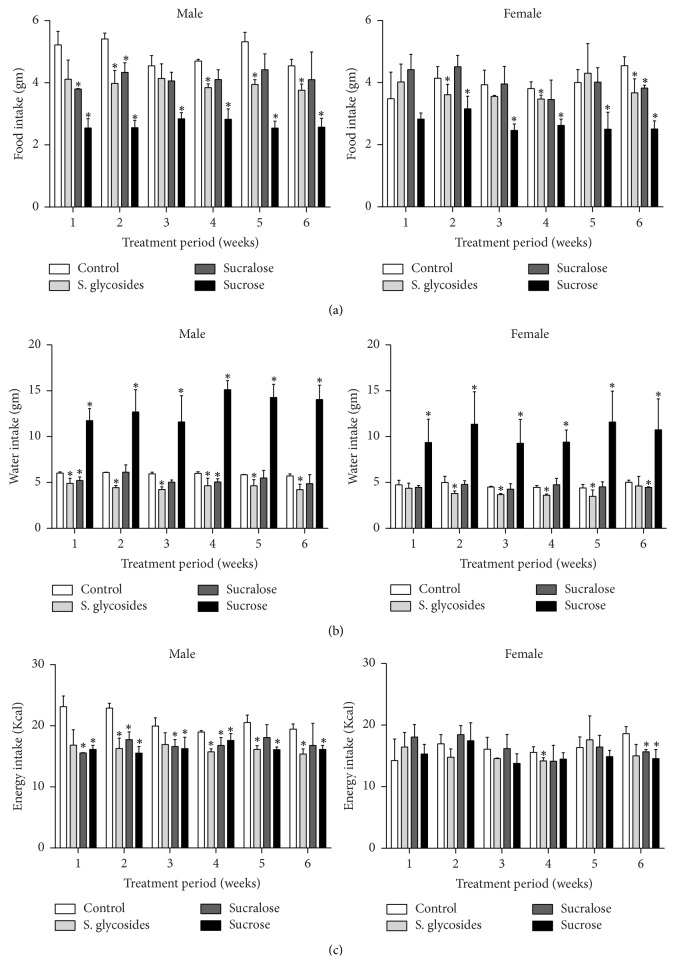
*Feeding behavior and energy intake of sweetener-supplemented mice.* 8-week old male and female mice were supplemented with commercial sweeteners in their drinking water for 6 weeks. Mean weekly intake was calculated from daily water and food consumption by 3 animals per cage. Mean weekly food (a) and water intake (b) for male and female mice are shown. Total energy intake (c) was also calculated from food and water intake and presented as weekly energy intake (Kcal) for male and female mice. Data are presented as mean ± SEM. ^*∗*^*p* < 0.05 compared to control group. *n* = 9 male and 9 female animals per group.

In order to evaluate the effect of commercial sweetener supplementation on feeding behavior, we determined daily food and water intake in our experimental animals over the 6-week treatment period. Our results show a significant decrease in food intake, with a corresponding increase in water intake, in both male and female mice from the sucrose group, compared to controls (at week 6, 2.57 ± 0.28 versus 4.55 ± 0.21 and 14.05 ± 1.56 versus 5.71 ± 0.22 for food and water intake, resp., in males and 2.50 ± 0.25 versus 4.54 ± 0.28 and 10.75 ± 3.3 versus 5.02 ± 0.24 for food and water intake, resp., in females, *p* < 0.05) (Table 1, Figures 1(a) and 1(b)). Preference for either sucrose- or nonnutritive sweetener-supplemented beverages is highly variable and has been previously described, with animals preferring either plain water, sucrose-sweetened water, or water supplemented with sucralose under different conditions [21–24].

In contrast to sucrose, nonnutritive sweetener-supplemented animals presented differential alterations in their feeding behavior, with the SG group showing the most significant effect. SG-supplemented male mice presented a significant sustained reduction in both food and water intake throughout the study period, compared to controls (at week 6, 3.77 ± 0.19 versus 4.55 ± 0.21 for food intake and 4.22 ± 0.66 versus 5.71 ± 0.22 for water intake, *p* < 0.05) (Table 1, Figures 1(a) and 1(b)). SG-supplemented females presented a similar effect on water intake, which returned to normal at the end of the study period (week 2, 3.8 ± 0.27 versus 4.99 ± 0.68, *p* < 0.05; week 6, 4.62 ± 1.03 versus 5.02 ± 0.24), while significant reductions on food intake were observed in these animals at weeks 2, 4, and 6 (*p* < 0.05) (Table 1, Figures 1(a) and 1(b)). On the other hand, sucralose-supplemented mice show only minor alterations in feeding behavior compared to control animals, with sucralose-supplemented male mice having a significant decrease in food and water intake only at the start of treatment (3.80 ± 0.01 versus 5.22 ± 0.43, *p* < 0.05, for food intake; 5.20 ± 0.4 versus 6.02 ± 0.14, *p* < 0.05 for water intake), later returning to similar levels as those from control animals, while female mice from the same group showed only a decrease in food intake at the end of treatment (3.82 ± 0.09 versus 4.54 ± 0.28, for food intake; 4.45 ± 0.06 versus 5.02 ± 0.24 for water intake, *p* < 0.05) (Table 1, Figures 1(a) and 1(b)). Thus, our data suggest that SG supplementation downregulates feeding behavior preferentially in male mice.

### 3.2. Changes in Total Energy Intake in Sweetener-Supplemented Mice

Total energy intake was also calculated from food and water consumption values, in order to evaluate the impact of feeding behavior on total calorie consumption. In male animals, significant decreases in total energy intake were observed in SG- and sucrose-supplemented groups, compared to controls, throughout the study, while sucralose induced a significant decrease in energy intake only in the first four weeks of treatment (Table 1). There were no significant differences among sweetener-supplemented male groups (Table 1, Figure 1(a)). In contrast, female mice did not show a clear pattern, as sweetener-supplemented groups presented increased energy intake during the first week of study (14.24 ± 3.5 Kcal for controls versus 16.45 ± 2.35 Kcal, 18.07 ± 2.01 Kcal, and 15.32 ± 1.56 Kcal for SG, sucralose, and sucrose, resp.), while later decreasing and becoming significantly lower than controls by the end of treatment in both the sucrose and sucralose groups, but not SG (14.56 ± 1.47 Kcal and 15.66 ± 0.36 Kcal, sucrose and sucralose, resp., versus 18.61 ± 1.16 Kcal for controls, *p* < 0.05) (Table 1, Figure 1(c)). Therefore, our results suggest that SG supplementation decreases total energy intake from diet in male mice only.

### 3.3. Differential Effects on Body Weight and Adiposity in Sweetener-Supplemented Mice

**Figure 2:**
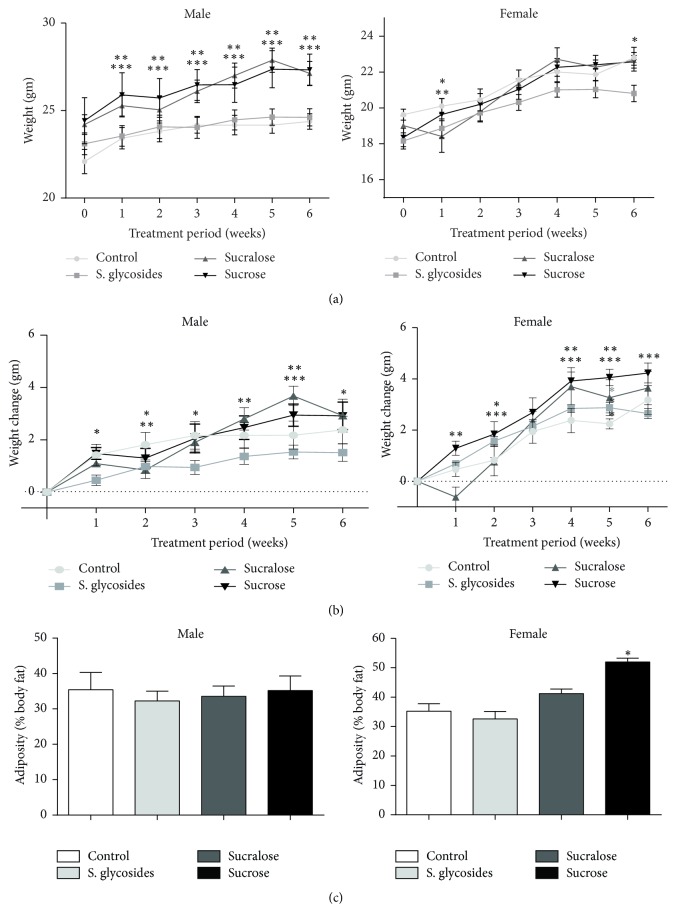
*Changes in body weight and composition in sweetener-supplemented mice. *Body weight was monitored on a weekly basis, and a single bioimpedance test was performed at the end of the 6-week supplementation period to assess body composition. Total weight of male and female animals (a), as well as cumulative weight gain of male and female mice (b), is shown. Data are presented as mean ± SEM. ^*∗*^*p* < 0.05 SG compared to control; ^*∗∗*^*p* < 0.05 sucrose compared to control and ^*∗∗∗*^*p* < 0.05 sucralose compared to control. Body fat percentage is shown (c) at the end of the study for male and female mice, respectively. Data are presented as mean ± SEM. ^*∗*^*p* < 0.05 compared to control. *n* = 9 male and 9 female/group.

To determine a possible impact of commercial sweetener supplementation on body weight and adiposity, we performed weekly measurements of body weight during the study period and assessed adiposity as % body fat using a single bioimpedance test at the end of the study. Our results show that male mice from both the sucrose and sucralose groups presented the highest body weight throughout the study, being significantly different from both control and SG-supplemented animals by the end of the 6-week supplementation period (27.33 ± 0.9 g and 27.12 ± 0.67 g, sucrose and sucralose versus 24.38 ± 0.44 g and 24.61 ± 0.49 g, controls and SG, resp.; *p* < 0.05) (Table 2, Figure 2(a)). In female groups, the only significant difference was observed in SG-supplemented females, which had lower weight at week 2 and at the end of the study period, compared to the control group (20.81 ± 0.46 g versus 22.81 ± 0.58 g. *p* < 0.05) (Table 2, Figure 2(a)).

Since not all animals presented the same weight at the beginning of the study, we evaluated differences in weight gain to determine the actual increase in body mass. Consistently with results from body weight, our data on weight gain showed that male mice from the SG group gained less weight compared to controls throughout the study period (at week 6, 1.51 ± 0.33 g versus 2.38 ± 0.52 g. *p* < 0.05) (Table 2, Figure 2(b)). Since weight was higher in SG mice compared to controls at the start of the study, both groups have similar weight at the end (Figure 2(a)). In contrast, sucralose-supplemented animals show an initial decrease in weight gain and end up gaining significantly more weight than controls by week 5 (3.67 ± 0.37 g versus 2.17 ± 0.54 g, *p* < 0.05) (Table 2, Figure 2(b)). For female animals, both sucrose and sucralose supplementation appear to significantly increase weight gain compared to controls during the study period. By week 6, sucrose supplementation induced a greater weight gain compared to the control group (4.23 ± 0.39 g versus 3.17 ± 0.56 g, *p* < 0.05), while sucralose-supplemented mice had gained more weight by week 4 (3.7 ± 0.74 g versus 2.37 ± 0.47 g, *p* < 0.05) and week 5 (3.26 ± 0.69 g versus 2.24 ± 0.19 g. *p* < 0.05), maintaining a tendency for greater weight gain by week 6 (3.64 ± 0.64 g versus 3.17 ± 0.56 g), although, similarly to male animals, there is an initial decrease in weight gain in sucralose-supplemented females that is compensated with a significant increase later on (Figure 2(b)). No significant differences were observed in SG-supplemented females compared to controls (2.65 ± 0.19 g versus 3.17 ± 0.56 g by week 6). The significant difference in body weight previously observed between controls and SG-supplemented animals probably stems from the fact that females in the SG group had a lower weight at the start of the study (Figure 2(a)). Thus, our results suggest that SG supplementation decreases, while sucralose appears to increase, weight gain in our experimental animals.

Regarding adiposity, no significant differences were found in body fat mass among groups in male mice, whereas female mice from the sucrose group had significantly increased adiposity compared to controls (51.95 ± 1.33% versus 35.23 ± 2.54%, resp.; *p* < 0.05) (Figure 2(c)). A nonsignificant tendency towards increased adiposity was also observed in female mice from the sucralose group (Figure 2(c)).

### 3.4. Alterations in the Expression of JAK2/STAT3 Pathway Proteins in the Brain of Sweetener-Supplemented Mice

**Figure 3:**
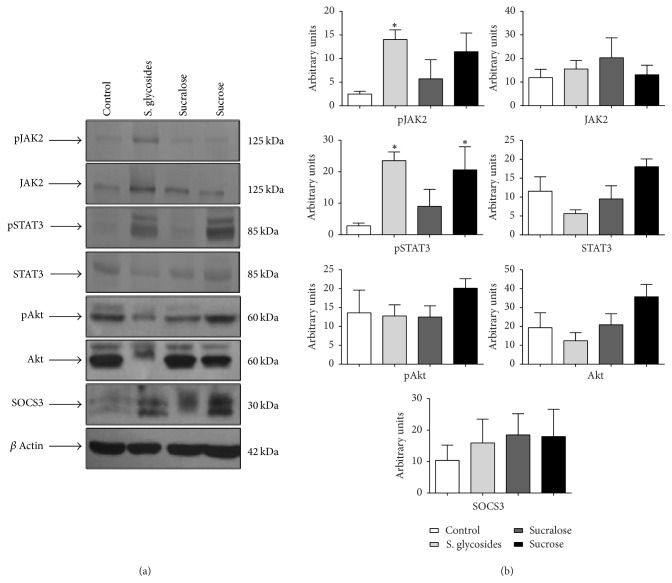
*JAK2/STAT3, Akt, and SOCS expression in the brains of sweetener-supplemented male mice. *Total brain protein extracts obtained at the end of the study were used to assess total and phosphorylated JAK2, STAT3, and Akt, as well as SOCS3, in male mice by western blot. *β*-actin was used as a loading control (a). *n* = 6 male animals per group. One representative experiment of six is shown. (b) Densitometry analysis (average of six different experiments), expressed in mean of arbitrary units ± SEM. One-way analysis of variance was performed. ^*∗*^*p* < 0.05 compared to control group.

To establish the possible effect of sweetener supplementation on a signaling pathway involved in the regulation of appetite and energy balance in the organism, the JAK2/STAT3 pathway, we analyzed the expression of total and phosphorylated forms of JAK2 and STAT3 in brain tissue from sweetener-supplemented mice. Densitometry analysis of protein bands obtained by western blotting from brain extracts shows significantly increased phosphorylation of both JAK2 and STAT3 in the brain of male mice from the SG group, compared to controls (14.02 ± 2.04 versus 2.49 ± 0.58 AU for pJAK2 and 23.5 ± 2.77 versus 2.81 ± 0.88 AU for pSTAT3, resp.; *p* < 0.05) (Figures 3(a) and 3(b)). pSTAT3, but not pJAK2, was also significantly increased in the brain of sucrose-supplemented male mice compared to controls (20.58 ± 7.34 AU versus 2.81 ± 0.88 AU; *p* < 0.05) (Figures 3(a) and 3(b)). In contrast, expression of total JAK2 and STAT3 in male mice was not significantly different among groups (Figures 3(a) and 3(b)).

**Figure 4:**
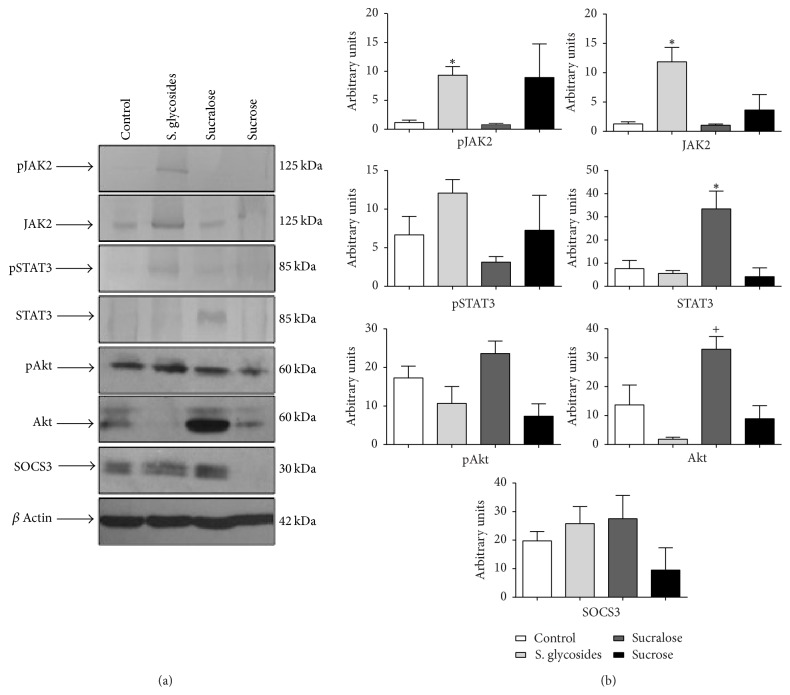
*JAK2/STAT3, Akt and SOCS expression in the brains of sweetener-supplemented female mice. *Total brain protein extracts obtained at the end of the study were used to assess total and phosphorylated JAK2, STAT3 and Akt, as well as SOCS3, in female mice by western blot. *β*-actin was used as a loading control (a). *n* = 6 female animals per group. One representative experiment of six is shown. (b) Densitometry analysis (average of six different experiments), expressed in mean of arbitrary units ± SEM. One-way analysis of variance was performed. ^*∗*^*p* < 0.05 compared to control group. ^+^*p* < 0.05 sucralose compared to SG group.

On the other hand, female mice showed upregulated JAK2 and pJAK2 expression when supplemented with SG (11.85 ± 2.45 versus 1.27 ± 0.34 AU for JAK, and 9.34 ± 1.5 versus 1.16 ± 0.4 AU for pJAK2. *p* < 0.05), as well as increased expression of STAT3 when supplemented with sucralose, compared to controls (33.44 ± 7.68 versus 7.6 ± 3.6 AU, resp.) (Figures 4(a) and 3(b)).

In addition to JAK2 and STAT3, we analyzed the expression of total and phosphorylated forms of Akt, as it is a key molecule in the regulation of STAT3 activity through the functions of FOXO1, one of the main STAT3 negative regulators [25]. Our results show no significant differences in either total or phosphorylated Akt expression among groups, in either male or female mice, even though we observed a trend towards decreased Akt expression in all mice from the SG group (Figures 3(b) and 4(b)). A statistically significant difference in total Akt expression was found between the SG and sucralose female groups (*p* < 0.05) (Figure 4). Analysis of phosphorylated/total protein ratios was also performed. As shown in supplementary Figure 1, we found a statistically significant difference in pJAK2/JAK2 ratio in male mice from the SG group, compared to controls (*p* < 0.05). We also observed differences between the sucralose and SG groups in females for both pSTAT3/STAT3 and pAtk/Akt ratios (*p* < 0.05) (Sup. Figure 1).

Finally, we analyzed the expression of SOCS3, one of the most important negative regulators for the JAK2/STAT3 pathway, in the brain of our experimental animals. We found no significant differences in SOCS3 expression among groups in either male or female mice in our study (Figures 3 and 4).

### 3.5. ObRb Leptin Receptor Expression in the Brain of Sweetener-Supplemented Mice

**Figure 5:**
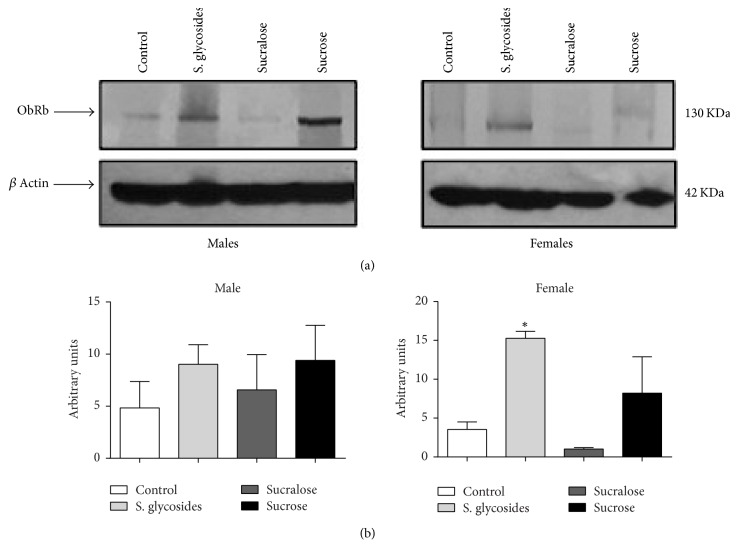
*ObRb expression in the brains of sweetener-supplemented mice.* Total brain protein extracts were obtained at the end of the supplementation period and ObRb expression was assessed in male and female mice by western blot (a). *β*-actin expression was used as a loading control. One representative experiment of six is shown. Densitometry analysis was performed (average of six experiments) and is expressed in mean of arbitrary units ± SEM in graphs for male and female mice (b). *n* = 6 male and 6 female animals per group. One-way analysis of variance was performed. ^*∗*^*p* < 0.05 compared to control group.

In addition to the evaluation of the signaling pathway, we sought to determine if there were any differences in the expression of the ObRb receptor in the brain of our experimental animals, as leptin binding to this receptor activates JAK2/STAT3 signaling to inhibit appetite and increase energy expenditure in the organism. Densitometry analysis revealed no significant difference in brain ObRb expression among groups in male mice, although we observed a nonsignificant trend towards increased expression in all sweetener-supplemented groups compared to controls (Figures 5(a) and 5(b)). In contrast, female mice from the SG group showed significantly increased expression of ObRb compared to the control group (15.28 ± 0.89 versus 3.53 ± 0.96 AU, resp.; *p* < 0.05), while the sucralose group showed a nonsignificant decrease (Figures 5(a) and 5(b)).

**Figure 6:**
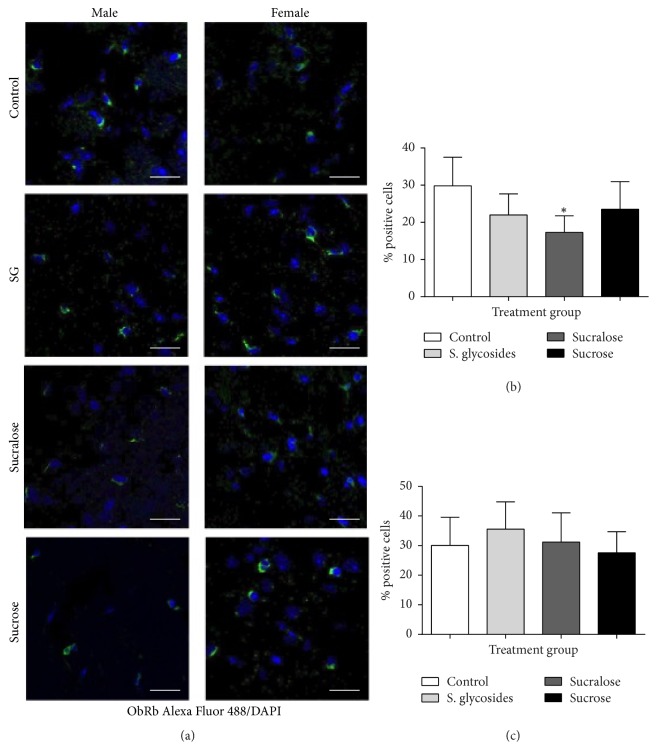
*Relative frequency of ObRb+ cells in brain tissue sections from sweetener-supplemented mice. *10 *μ*m coronal brain sections were obtained at the end of the supplementation period. Slides were then stained with anti-ObRb and secondary Alexa 488-conjugated antibody and observed in a confocal microscope. (a) Expression of ObRb/A488 (green) and cell nuclei in the brains of male and female mice. The frequency of ObRb-positive cells per sample is shown in (b) (males) and (c) (females). *n* = 3 male and 3 female animals per group; 260–470 cells were counted by group. One-way ANOVA was performed. ^*∗*^*p* < 0.05 compared to control group.

We also observed the expression of ObRb directly in brain tissue by immunofluorescence (Figure 6(a)). Our results show a significant difference in the frequency of ObRb+ cells only in male mice supplemented with sucralose, compared to controls (17.29 ± 4.47% versus 29.86 ± 7.7%, resp.; Figure 6(b)). No significant differences with controls were observed in other male or female groups; however, we also observed significant differences between sucralose-supplemented male and female mice (17.29 ± 4.47% versus 31.2 ± 9.87%), but no significant difference was found between SG groups. Negative controls for confocal microscopy were performed by staining cells with the A488 secondary antibody in the absence of primary antibody (Sup.  Figure 2).

## 4. Discussion

Sweetener consumption is a common practice in the general population as a means to decrease calorie intake from diet; however, accumulating evidence has challenged the assumption that nonnutritive sweeteners favor weight loss and aid in the control of appetite in people needing to reduce sugar intake [5, 25–28]. In the present study, we evaluated the effect of chronic intake of commercial sweeteners on brain signaling pathways related to the control of appetite and energy balance.

For our experimental model we chose BALB/c mice, as they are a well-characterized strain for the study of both the nervous and immune systems and it has been used in our research projects for a long time. Although BALB/c mice may be considered as less sensitive to the taste of specific nonnutritive sweeteners than other mouse strains [29–31], they have well-characterized responses to the sweet taste of fructose [32], demonstrating sweet sensitivity in appropriate conditions. Additionally it has been demonstrated that nonnutritive sweeteners like SG have direct physiologic effects on this mouse strain, regardless of taste [33, 34]. Since our study did not involve comparison of taste preference among our experimental animals, and since most studies on the effects of nonnutritive sweetener supplementation have been performed on the C57BL/6 strain, we decided to analyze our experimental variables on BALB/c mice.

Similarly, most studies on the effects of nonnutritive sweetener supplementation are based on the use of purified versions of the relevant sweeteners at different concentrations to determine their physiological impact. We decided to use commercially available versions of nonnutritive sweeteners freely available to the general population, as these products are the most commonly used form to add sweet flavor to food used voluntarily by humans and, so, represent the usual way in which these compounds become a part of an individual's regular diet. Our belief is that use of commercial presentations of nonnutritive sweeteners may provide a more accurate representation of the way in which nonnutritive sweeteners enter the organism and, thus, would allow us to better determine their possible physiological effects on a living organism.

Although sweet taste preference is a highly variable condition in mice, our results show an expected increase in water intake, followed by a corresponding decrease in feeding, in the sucrose-supplemented group, as sugar-sweetened water is preferred by mice under specific study conditions, an effect that is dependent on glucose rather than fructose content [35–39]. In contrast, although supplementation with sucralose did not appear to have a significant effect on feeding behavior, we found a significant decrease in food and water intake in mice supplemented with SG, an effect that was particularly evident in male mice.

Decreased water intake may be dependent on taste preference, as previous studies have reported differential effects of sweetener supplementation on feeding behavior, such as avoidance of sucralose in rats under specific circumstances [40], while SG-sweetened beverages are reported to be preferred by specific mouse and rat strains, stimulating overdrinking compared to plain water in mice, but not in rats, under the same experimental conditions [41]. However, we found that in our model, SG-supplemented animals consumed significantly less water than controls, suggesting that sweetener concentrations used, as well as the effect of any additives present in their commercial presentation, could cause an aversive reaction that limits the amount of fluid intake by our experimental animals. Decreased preference for nonnutritive sweeteners has been previously observed in BALB/c mice when compared to other mouse strains, particularly C57BL/6 mice, which tend to show increased preference to nonnutritive sweetener-supplemented water [29–31]. However, it should be noted that those studies use purified nonnutritive sweeteners as supplements, whereas our study relies on the use of commercially available preparations of these compounds, which include some additives such as maltodextrin, which have been reported to affect taste preference and feeding behavior in other animal models [42]; therefore, there is no direct comparison for the results on taste preference observed in our study. Similarly, these studies do not take into account any potential differences that may be present in taste preference among male and female mice, an effect that is clearly shown in our results and which should be accounted for in sweetener supplementation study design. Nevertheless, regardless of taste preference, our animals still consumed a significant amount of sweetener-supplemented water daily, allowing us to continue our analysis on the effects of frequent sweetener intake* in vivo*.

Data on food and water intake are also consistent with the observed decrease in total energy uptake in SG-supplemented male mice, as we observed that SG supplementation reduces both water and food intake in this group and therefore would be expected to reduce energy availability from diet. Accordingly, we also observed decreased weight gain in SG-supplemented male mice, whereas female mice supplemented with sucrose show increased adiposity and weight gain. However, we did not observe an overall increase in energy uptake in sucrose-supplemented mice, a result that is counterintuitive to the idea that sucrose supplementation provides additional calories to diet and should increase total energy intake over a relatively long period of time, suggesting additional factors regulating energy balance in our model.

Our findings contrast with murine models of sweetener supplementation using saccharin and acesulfame K, where increased food intake has been observed [2, 3]; however, since these sweeteners have been shown to promote a different set of metabolic changes in experimental animals, it is possible that the mechanisms that trigger the caloric balance deficits observed in saccharin and acesulfame K supplementation are different from those affected by sucralose and SG intake. In addition, there are reports of sex differences in weight gain in rats supplemented with saccharine, as well as weight gain in both male and female saccharine-supplemented animals fed a high energy content diet [43], suggesting that it is actually the combination of nonnutritive sweeteners and a high calorie diet that synergizes to induce weight gain under nonnutritive sweetener supplementation regimes.

Sweetener supplementation has also been shown to modify the release of gastrointestinal hormones like GLP-1, affecting glucose homeostasis and energy balance* in vivo *[44], further demonstrating that sweeteners may cause relevant metabolic effects by a variety of pathways. Furthermore, additional physiological factors, including expression and function of growth hormone and IGF-1, may be altered by frequent sweetener supplementation. These factors are known to affect lipid metabolism and adiposity in different animal models [45, 46], and their physiologic regulation should be studied under sweetener supplementation regimes. Finally, another possibility could be related to altered physical activity in our animals; however, since we did not perform systematic observations of physical activity in this study, we cannot provide adequate data on this subject.

Decreased appetite and weight gain in noncaloric sweetener-supplemented mice may be related to the activity of the JAK2/STAT3 signaling pathway, as our results show increased expression of the phosphorylated form pJAK2 in both male and female mice supplemented with SG, as well as an increased pJAK2/JAK2 ratio in male SG mice, while pSTAT3 was also increased in male SG-supplemented mice and there was a significant difference in pSTAT3/STAT3 and pAkt/Akt ratios among female mice from the SG and sucralose groups, corresponding with differences observed in their feeding behavior and weight gain.

Upregulated pJAK2 and pSTAT3 signaling in our model could correspond with the reduced feeding behavior seen in SG-supplemented mice, as STAT3 signaling is known to promote POMC expression, which is then cleaved into *α*-MSH, a potent anorectic peptide [12, 13]. In addition, we did not observe increased expression of Akt, pAkt, or SOCS3 in sweetener-supplemented mice, while detecting a nonsignificant trend towards decreased Akt expression in SG-supplemented mice. As these molecules are involved in the negative regulation of the JAK2/STAT3 signaling pathway, absence of upregulation of these molecules in our model could favor the activity of the JAK2/STAT3 pathway and its anorectic effects. Therefore, our results suggest that SG supplementation promotes increased JAK2/STAT3 signaling in the brain, inhibiting appetite. Further studies should focus on analyzing the expression and activity of POMC and *α*-MSH in the brain after sweetener supplementation, as well as the activity of other negative regulators of the JAK2/STAT3 signaling pathway, such as PTP1B and insulin receptor substrate-1 (IRS-1) [47]. Despite our results regarding the signaling pathways, it is important to point out that a great limitation for our study is the fact that we used total brain proteins instead of proteins isolated from hypothalamus, mostly due to technical limitations and the fact that BALB/c mice brains are quite small, and the amount of proteins obtained from the hypothalamus was not enough to perform a complete western blot analysis.

Since the anorectic activity of the JAK2/STAT3 signaling pathway is related to the functions of leptin, one of the most relevant and well-studied hormones involved in the control of appetite and energy balance in the organism, it is important to determine the effect of sweetener supplementation on leptin-dependent signaling, particularly through the long isoform ObRb receptor. Our results showed a relevant increase in the frequency of ObRb+ cells in the brain of SG-supplemented female mice, corresponding with the observed increase in pJAK2 in the same animals; however, no significant difference was observed in male sweetener-supplemented mice, although we observed a trend towards increased frequency of ObRb+ cells in these animals in all sweetener-supplemented groups compared to controls. In contrast, immunofluorescence analysis showed a significant decrease in the number of ObRb+ cells in the male sucralose group, although this decrease was not evident in the western blot. This discrepancy may be related to the fact that immunofluorescence analysis is representative of a very limited number of cells within the brain, while protein expression analysis is based on total protein expression in the whole brain. In this sense, immunofluorescence analysis focused on specific brain areas, such as the hypothalamus, would be most relevant. ObRb is essential for initial leptin-dependent signaling in cells, and decreased expression or function of this receptor has been found to elicit a hyperphagic and obese phenotype [17].

Collectively, our results suggest that, in contrast to sucrose, chronic intake of the nonnutritive sweetener SG downregulates feeding behavior, as well as total energy intake, promoting a decrease in body weight in mice, whereas sucralose did not have the same effect. The effect of SG on appetite and weight gain in our model is likely dependent on increased activity of the JAK2/STAT3 signaling pathway in the brain and may be related to leptin-dependent signaling through the ObRb receptor, as we found significant sex-dependent increases in pJAK2/JAK2, pSTAT3/STAT3 and pAkt/Akt ratios and frequency of ObRb+ cells in the brain of male and female animals from nonnutritive sweetener-supplemented groups. Correspondingly, sweetener supplementation shows diverse effects on male and female mice, as females supplemented with sucrose gained more weight and had increased adiposity compared to their male counterparts, an effect that may be related to differences in metabolic rates related to hormonal variations among male and female mice [48].

Our results suggest that chronic intake of commercial sweeteners, particularly SG, modifies the activity of brain signaling pathways related to the control of appetite and energy balance in the organism. While the observed effect cannot be directly attributed to the noncaloric sweetener alone, since commercial nonnutritive sweetener formulations contain additives like maltodextrin, the fact that chronic supplementation with these mixtures is capable of inducing changes in brain signaling pathways is highly relevant, as these are the type of additives that are commonly used by the general population worldwide, instead of purified nonnutritive sweeteners alone. Thus, despite the fact that our study does not allow us to specifically relate the observed results to the effect of the nonnutritive sweetener by itself, the alterations reported in this study provide evidence that frequent intake of commercially available nonnutritive sweetener formulations promotes modifications in feeding behavior, energy intake, and body composition, with gender being a determinant factor that should be considered in future study designs. Considering that sweetener intake is widespread among the general population, our study provides further evidence suggesting these compounds may alter metabolic pathways that are relevant to the control of appetite and energy balance* in vivo* and, thus, warrant further study to determine their possible beneficial or detrimental effects on human health.

## Figures and Tables

**Table 1:** *Food, water, and energy intake*. Data are expressed as mean ± SEM. ^*∗*^*p* < 0.05 compared to control group in each week (differences between means with a 95% confidence interval). *n* = 9 male and 9 female animals per group.

Week	Group	Food intake (g)	Water intake (g)	Energy intake (Kcal)	Food intake (g)	Water intake (g)	Energy intake (Kcal)
		Male			Female		
1	Control	5.22 ± 0.43	6.02 ± 0.14	23.13 ± 1.76	3.48 ± 0.85	4.74 ± 0.50	14.24 ± 3.50
	SG	4.12 ± 0.62	4.91 ± 0.54^**∗**^	16.83 ± 1.34	4.02 ± 0.57	4.37 ± 0.55	16.45 ± 2.35
	Sucralose	3.80 ± 0.01^**∗**^	5.20 ± 0.4^**∗**^	15.55 ± 0.06^**∗**^	4.41 ± 0.49	4.47 ± 0.21	18.07 ± 2.01
	Sucrose	2.6 ± 0.3^**∗**^	11.75 ± 1.31^**∗**^	16.13 ± 0.69^**∗**^	2.83 ± 0.19	9.36 ± 2.55^**∗**^	15.32 ± 1.56

2	Control	5.41 ± 0.18	6.08 ± 0.03	22.91 ± 0.77	4.14 ± 0.37	4.99 ± 0.68	16.95 ± 1.51
	SG	3.99 ± 0.41^**∗**^	4.44 ± 0.22^**∗**^	16.30 ± 1.68^**∗**^	3.61 ± 0.33^**∗**^	3.8 ± 0.27^**∗**^	14.78 ± 1.35
	Sucralose	4.33 ± 0.31^**∗**^	6.12 ± 0.79	17.73 ± 1.28^**∗**^	4.51 ± 0.36	4.78 ± 0.39	18.46 ± 1.49
	Sucrose	2.55 ± 0.23^**∗**^	12.7 ± 2.43^**∗**^	15.54 ± 1.07^**∗**^	3.16 ± 0.40^**∗**^	11.36 ± 3.5^**∗**^	17.47 ± 2.91

3	Control	4.55 ± 0.33	5.94 ± 0.18	19.96 ± 1.36	3.93 ± 0.47	4.51 ± 0.08	16.08 ± 1.92
	SG	4.14 ± 0.47	4.24 ± 0.28^**∗**^	16.94 ± 1.92	3.55 ± 0.02	3.67 ± 0.13^**∗**^	14.55 ± 0.11
	Sucralose	4.06 ± 0.28	5.05 ± 0.23	16.61 ± 1.14^**∗**^	3.95 ± 0.56	4.27 ± 0.59	16.18 ± 2.31
	Sucrose	2.84 ± 0.19^**∗**^	11.61 ± 2.86^**∗**^	16.27 ± 1.87^**∗**^	2.46 ± 0.20^**∗**^	9.27 ± 2.5^**∗**^	13.78 ± 1.54

4	Control	4.70 ± 0.06	5.99 ± 0.18	18.99 ± 0.23	3.80 ± 0.22	4.46 ± 0.20	15.57 ± 0.90
	SG	3.84 ± 0.12^**∗**^	4.63 ± 0.82^**∗**^	15.73 ± 0.49^**∗**^	3.47 ± 0.12^**∗**^	3.6 ± 0.14^**∗**^	14.20 ± 0.52^**∗**^
	Sucralose	4.10 ± 0.31	5.05 ± 0.35^**∗**^	16.80 ± 1.3^**∗**^	3.45 ± 0.62	4.75 ± 0.68	14.13 ± 2.5
	Sucrose	2.82 ± 0.33^**∗**^	15.12 ± 0.98^**∗**^	17.62 ± 1.11^**∗**^	2.62 ± 0.20^**∗**^	9.4 ± 1.32^**∗**^	14.49 ± 1.01

5	Control	5.32 ± 0.30	5.84 ± 0.03	20.53 ± 1.23	4.00 ± 0.42	4.4 ± 0.37	16.37 ± 1.72
	SG	3.95 ± 0.15^**∗**^	4.64 ± 0.66^**∗**^	16.15 ± 0.62^**∗**^	4.3 ± 0.95	3.49 ± 0.70^**∗**^	17.61 ± 3.9
	Sucralose	4.42 ± 0.51	5.5 ± 0.80	18.09 ± 2.09	4.01 ± 0.46	4.53 ± 0.54	16.43 ± 1.9
	Sucrose	2.54 ± 0.22^**∗**^	14.27 ± 1.44^**∗**^	16.10 ± 0.43^**∗**^	2.50 ± 0.54^**∗**^	11.6 ± 3.36^**∗**^	14.88 ± 1.02

6	Control	4.55 ± 0.21	5.71 ± 0.22	19.46 ± 0.85	4.54 ± 0.28	5.02 ± 0.24	18.61 ± 1.16
	SG	3.77 ± 0.19^**∗**^	4.22 ± 0.66^**∗**^	15.39 ± 0.80^**∗**^	3.6 ± 0.44^**∗**^	4.62 ± 1.03	15.02 ± 1.82
	Sucralose	4.1 ± 0.88	4.88 ± 0.98	16.79 ± 2.09	3.82 ± 0.09^**∗**^	4.45 ± 0.06^**∗**^	15.66 ± 0.36^**∗**^
	Sucrose	2.57 ± 0.28^**∗**^	14.05 ± 1.56^**∗**^	16.14 ± 0.65^**∗**^	2.50 ± 0.25^**∗**^	10.75 ± 3.3^**∗**^	14.56 ± 1.47^**∗**^

**Table 2:** *Weight and weight change per weeks of treatment.* Data are expressed as mean ± SEM. ^*∗*^*p* < 0.05 compared to control group in each week (differences between means with a 95% confidence interval).  *n* = 9 male and 9 female animals per group.

Week	Group	Weight (gm)	Weight change (gm)	Weight (gm)	Weight change (gm)
		Male		Female	
0	Control	22.00 ± 0.69	/	19.63 ± 0.31	/
	SG	23.1 ± 0.62	/	18.16 ± 0.45	/
	Sucralose	24.2 ± 0.56	/	19.02 ± 0.54	/
	Sucrose	24.42 ± 1.32	/	18.35 ± 0.51	/

1	Control	23.42 ± 0.61	1.42 ± 0.41	20.1 ± 0.42	0.46 ± 0.28
	SG	23.55 ± 0.59	0.45 ± 0.20^*∗*^	18.86 ± 0.53^*∗*^	0.68 ± 0.12
	Sucralose	25.28 ± 0.59^*∗*^	1.08 ± 0.11	18.41 ± 0.88^*∗*^	−0.61 ± 0.39^*∗*^
	Sucrose	25.9 ± 1.26^*∗*^	1.47 ± 0.22	19.64 ± 0.55	1.28 ± 0.27

2	Control	23.81 ± 0.60	1.81 ± 0.46	20.45 ± 0.61	0.82 ± 0.61
	SG	24.07 ± 0.67	0.97 ± 0.25^*∗*^	19.73 ± 0.45	1.56 ± 0.18^*∗*^
	Sucralose	25.04 ± 0.76^*∗*^	0.84 ± 0.32^*∗*^	19.8 ± 0.58	0.77 ± 0.15
	Sucrose	25.72 ± 1.11^*∗*^	1.3 ± 0.38	20.2 ± 0.60	1.84 ± 0.49^*∗*^

3	Control	24.16 ± 0.45	2.16 ± 0.52	21.57 ± 0.55	1.94 ± 0.46
	SG	24.04 ± 0.63	0.94 ± 0.26^*∗*^	20.31 ± 0.44	2.14 ± 0.21
	Sucralose	26.10 ± 0.65^*∗*^	1.9 ± 0.33	21.37 ± 0.27	2.35 ± 0.36
	Sucrose	26.48 ± 0.88^*∗*^	2.05 ± 0.55	21.04 ± 0.55	2.68 ± 0.57

4	Control	24.17 ± 0.56	2.17 ± 0.48	22.01 ± 0.55	2.37 ± 0.47
	SG	24.47 ± 0.57	1.36 ± 0.30	21.01 ± 0.41	2.84 ± 0.15
	Sucralose	27 ± 0.71^*∗*^	2.8 ± 0.42^*∗*^	22.72 ± 0.64	3.7 ± 0.74^*∗*^
	Sucrose	26.48 ± 1.01^*∗*^	2.46 ± 0.45	22.27 ± 0.5	3.92 ± 0.35^*∗*^

5	Control	24.17 ± 0.47	2.17 ± 0.54	21.87 ± 0.33	2.24 ± 0.19
	SG	24.63 ± 0.47	1.53 ± 0.26	21.04 ± 0.47	2.87 ± 0.20
	Sucralose	27.87 ± 0.69^*∗*^	3.67 ± 0.37^*∗*^	22.28 ± 0.40	3.26 ± 0.69^*∗*^
	Sucrose	27.36 ± 1.06^*∗*^	2.94 ± 0.42^*∗*^	22.41 ± 0.53	4.05 ± 0.32^*∗*^

6	Control	24.38 ± 0.44	2.38 ± 0.52	22.81 ± 0.58	3.17 ± 0.56
	SG	24.61 ± 0.49	1.51 ± 0.33^*∗*^	20.81 ± 0.46^*∗*^	2.65 ± 0.19
	Sucralose	27.13 ± 0.67^*∗*^	2.92 ± 0.63	22.66 ± 0.27	3.64 ± 0.64
	Sucrose	27.33 ± 0.9^*∗*^	2.91 ± 0.53	22.58 ± 0.52	4.23 ± 0.39^*∗*^

## Abbreviations

*α*-MSH: Alpha-melanocyte-stimulating hormone
Akt: Protein kinase B
CNS: Central nervous system
JAK2: Janus kinase 2
Nhlh2: Nescient helix-loop-helix 2 transcription factor
ObRb: Isoform B of the leptin receptor
POMC: Proopiomelanocortin
SG: Steviol glycosides
STAT3: Signal transducer and activator of transcription 3
SOCS3: Suppressor of cytokine signaling 3
TRH: Thyrotropin releasing hormone.

## Supplementary Materials

Supplementary 1 Supplemental Figure 1:* densitometry analysis of phosphorylated/total proteins ratio*. Densitometry analysis (average of six different experiments) of *p*/total proteins ratio was performed and expressed in mean of arbitrary units ± SEM for male and female mice. One-way analysis of variance was performed. ^*∗*^*p* < 0.05 compared to control group. ^+^*p* < 0.05 sucralose compared to SG group.

Supplementary 2 Supplemental Figure  2:* negative control for Alexa 488 antibody*. 10 *μ*m coronal brain sections were obtained and slides were incubated with the Alexa 488-conjugated secondary antibody in the absence of primary antibody and then stained with DAPI to observe nuclei.
