# Supplementary material for: Chronic Intake of Commercial Sweeteners Induces Changes in Feeding Behavior and Signaling Pathways Related to the Control of Appetite in BALB/c Mice
Source: Biomed Res Int. 2018 Jan 28;2018:3628121. doi: 10.1155/2018/3628121 (PMC5896338; doi:10.1155/2018/3628121)
Supplement: Supplementary 1 — Supplemental Figure 1: densitometry analysis of phosphorylated/total proteins ratio. Densitometry analysis (average of six different experiments) of p/total proteins ratio was performed and expressed in mean of arbitrary units ± SEM for male and female mice. One-way analysis of variance was performed. ∗p < 0.05 compared to control group. +p < 0.05 sucralose compared to SG group. [file 3628121.f1.pptx]

## Slide 1
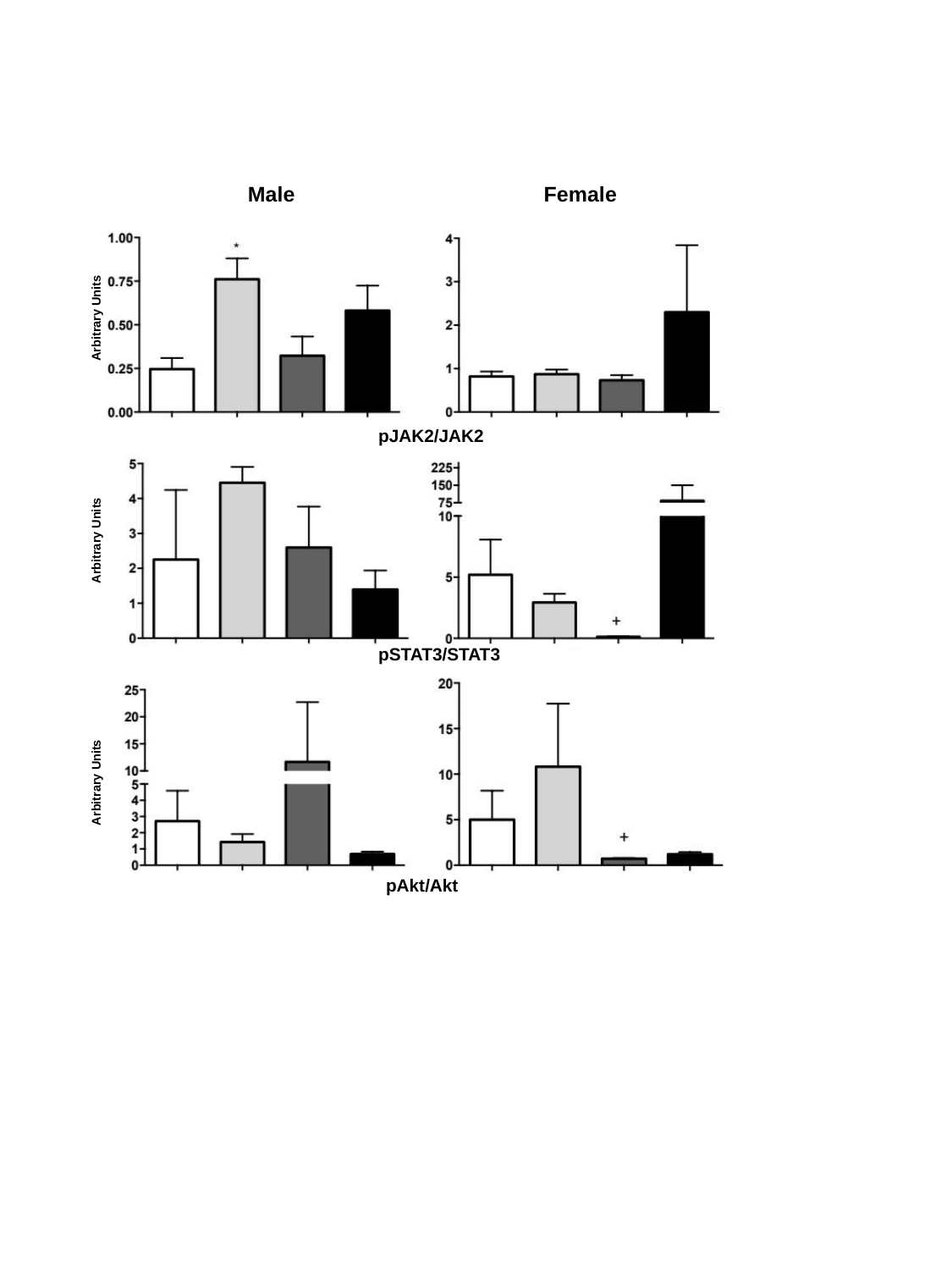

Male
Female
Arbitrary Units
pJAK2/JAK2
Arbitrary Units
pSTAT3/STAT3
Arbitrary Units
pAkt/Akt
