# Supplementary figures and images for: Chronic Intake of Commercial Sweeteners Induces Changes in Feeding Behavior and Signaling Pathways Related to the Control of Appetite in BALB/c Mice
Source: Biomed Res Int. 2018 Jan 28;2018:3628121. doi: 10.1155/2018/3628121 (PMC5896338; doi:10.1155/2018/3628121)

## Slide 1
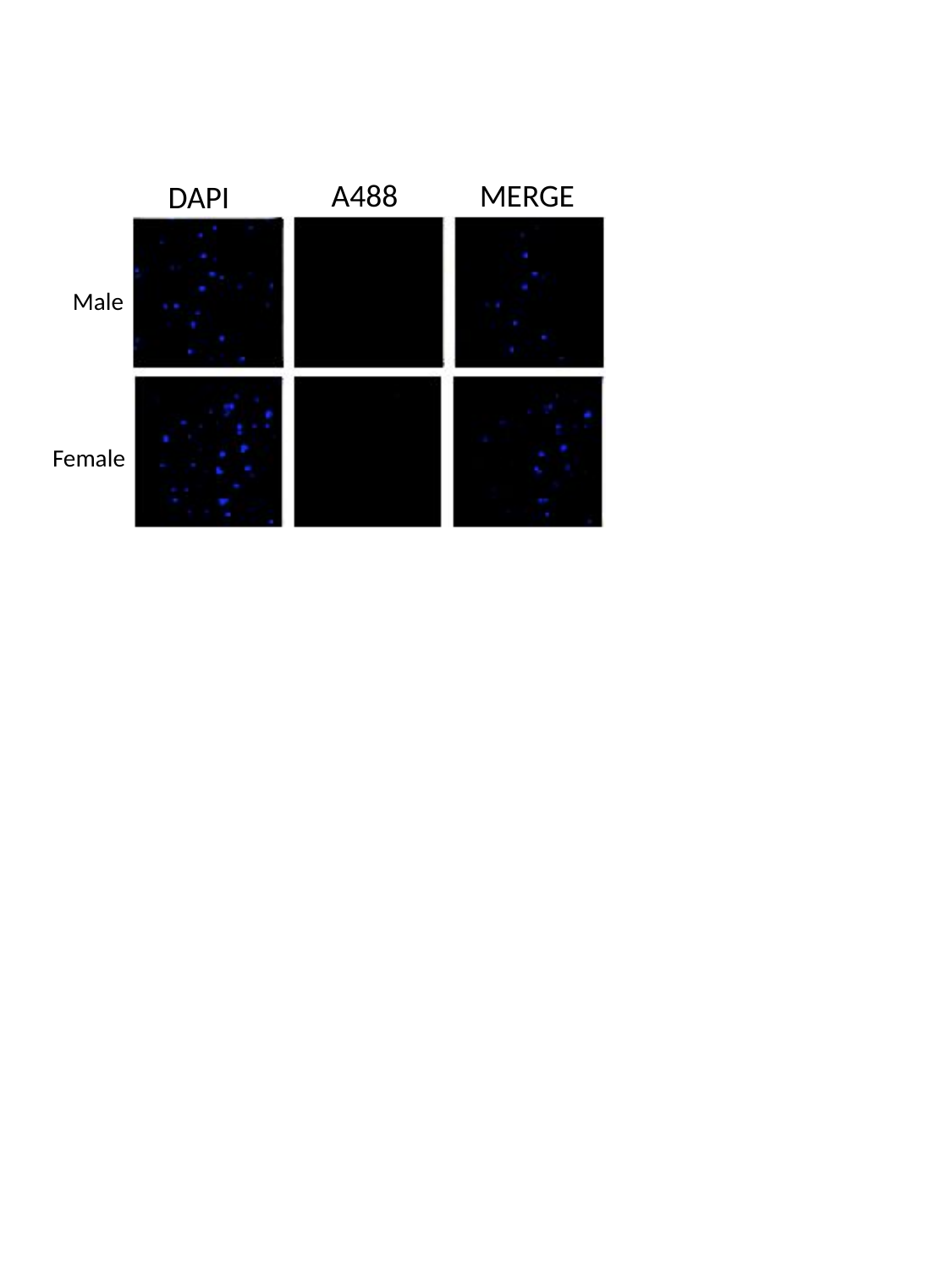

A488
MERGE
DAPI
Male
Female

Supplement: Supplementary 2 — Supplemental Figure 2: negative control for Alexa 488 antibody. 10 μm coronal brain sections were obtained and slides were incubated with the Alexa 488-conjugated secondary antibody in the absence of primary antibody and then stained with DAPI to observe nuclei. [file 3628121.f2.pptx]
